# Vascular considerations in autologous breast reconstruction using DIEP or MS-TRAM flaps: standard techniques, alternatives, and rescue strategies

**DOI:** 10.1515/iss-2025-0011

**Published:** 2025-05-22

**Authors:** Theresa Promny, Andreas Arkudas, Paula Huberth, Raymund E. Horch

**Affiliations:** Department of Plastic and Hand Surgery, University Hospital Erlangen, Friedrich-Alexander University Erlangen-Nürnberg (FAU), Erlangen, Germany

**Keywords:** supercharging, turbocharging, complications, flap revision, cross over bypass

## Abstract

**Objectives:**

Microsurgical breast reconstruction is an established method following mastectomy; however, various factors significantly influence the complication rate. This analysis evaluates the impact of vascular anastomosis techniques, previous surgeries, and radiotherapy on the success rate of microsurgical breast reconstruction.

**Methods:**

In a retrospective data analysis of 396 patients who underwent 447 flap procedures (single or bilateral microsurgical breast reconstruction) using tissue from the lower abdomen (either DIEP or MS-TRAM flaps), the type of venous and arterial anastomoses, the initial findings or surgical specifics at the thoracic recipient site and their influence on complications were examined.

**Results:**

Anastomosing two veins per flap did not result in a significant reduction of major complications. Flap loss was significantly more common when standard venous anastomosis to the internal mammary vein was not used, underscoring their role as the preferred recipient vessels. No major complications were observed in patients receiving a caudal anastomosis to the internal mammary artery. Patients with prior radiotherapy exhibited an increased need for arterial revisions. All documented seromas and infections occurred in irradiated patients.

**Conclusions:**

A differentiated surgical plan that considers optimal anastomosis techniques, supercharging strategies, and individual risk factors is crucial to reducing complications. Further studies are needed to optimize patient-specific strategies.

## Introduction

Microsurgical breast reconstruction is an established method for restoring the breast after mastectomy. Among the most widely used techniques are the deep inferior epigastric artery perforator (DIEP) flap and the muscle-sparing transverse rectus abdominis myocutaneous (MS-TRAM) flap [1]. Over the years, the internal mammary vessels (artery and vein) have become the gold standard for recipient vessels in autologous breast reconstruction due to their reliable caliber, location, and superior perfusion dynamics [2], 3]. However, in certain cases, alternative vessels such as the thoracodorsal artery and vein or the lateral thoracic vessels may be utilized, particularly if the internal mammary vessels are unavailable or compromised. Selecting the appropriate recipient vessels is critical, as vascular complications remain one of the most significant challenges in free flap breast reconstruction [4], [5], [6], [7]. The DIEP and MS-TRAM flap represent a reliable choice for breast reconstruction, with low rates of complications reported. However, vascular complications, including arterial thrombosis, venous congestion, and partial or total flap loss, can arise due to technical issues during microsurgical anastomosis, inadequate venous drainage, or pre-existing vascular damage. Venous congestion is the most frequently occurring perfusion-related complication, with reported incidence rates varying between 2 and 20 % [8], 9]. In cases with compromised deep veins, or inadequate venous outflow, alternative venous drainage strategies, such as dual venous anastomosis, supercharging, and utilizing alternative recipient veins, play a crucial role in preventing venous congestion and optimizing outcomes in DIEP/MS-TRAM flap breast reconstruction. Further, several factors increase the risk of vascular compromise at the recipient site, particularly the choice of vascular anastomoses, previous breast surgeries, and potential radiotherapy. General factors such as age, obesity, preoperative systemic or local treatments, such as chemotherapy or hormonal therapy, and comorbidities – including coagulation disorders – may also impact vascular health and wound healing [10], 11]. While the literature broadly acknowledges these general risk factors, detailed analyses focusing specifically on complications related to microsurgical anastomoses in the context of breast reconstruction remain limited. The aim of this study is to evaluate the impact of these factors on postoperative complications.

## Materials and methods

A retrospective data analysis was conducted on all patients at our institution – an academic center also responsible for training plastic surgeons – who underwent microsurgical breast reconstruction using tissue from the lower abdomen (either DIEP or MS-TRAM flaps) between January 2004 and December 2019 (n=424). The minimum follow-up period was 15 months.

Data were analyzed regarding potential complications at the thoracic recipient site. Major complications were defined as problems necessitating surgical intervention, while minor complications were events manageable through conservative treatment. Complications occurring at the recipient sites were separately studied. Major complications encompassed total or partial flap failure, the need for surgical revision due to arterial or venous insufficiency, postoperative hemorrhage, infection, or fluid accumulation (seroma). Even minimal wound healing impairment was documented and factored into the analysis of minor complications in addition to non-surgically treated wound dehiscence, localized infections, seroma formation, localized minimal flap necrosis. For the current investigation, we especially examined a possible correlation between anastomosis-related complications and associated risk factors and treatments, including surgical revisions and problem-solving approaches.

In particular, we analyzed the surgical approach and potential risk factors for complications related to venous anastomoses, the number of veins used, the type of venous connection in the cranial or caudal direction, and the use of interposition grafts. Additionally, we examined the initially performed supercharging of the superficial epigastric veins, the connection to the subclavian vein, the influence of in-flap anastomoses, as well as the arterial anastomosis situation at the internal mammary artery (with either caudal or cranial blood flow direction) and the use of arterial venous interposition grafts.

Categorical variables were evaluated using the Fisher’s exact test. Statistical analysis was conducted using GraphPad Prism 9 (GraphPad Software, San Diego, CA, USA). A p-Value≤0.05 was considered statistically significant.

## Results

During the analyzed treatment period, 424 patients underwent uni- or bi-lateral abdominal-based free flap breast reconstruction. 28 patients (29 free flaps) were excluded from evaluation since there were not sufficient data for the follow up, so that 447 flap procedures (single DIEP/MS-TRAM or double DIEP/MS-TRAM) performed in 396 patients were included. Patient demographic data is summarized in Table 1. Table 2 shows the distribution of major and minor complications at the recipient site. All vascular anastomosis scenarios were analyzed in detail.

**Table 1:** Demographics and patient characteristics.

Patients, n	396
Flaps, n	447
Age [years], mean ± SD (range)	52 ± 9 (30–77)
BMI [kg/m^2^], mean ± SD (range)	27 ± 4 (17–45)
Previous breast surgeries, n (%)	
– None (Simultaneous prophylactic mastectomy)	15 (3.4)
– Breast-conserving therapy	56 (12.5)
– Mastectomy	208 (46.5)
– Mastectomy + expander/prosthesis	156 (34.9)
– Mastectomy + earlier flap reconstruction	8 (1.8)
– Silicone prosthesis breast augmentation	4 (0.9)
Status after radiotherapy, n (%)	262 (59)

**Table 2:** Distribution of major and minor complications.

Major complication, n (%)	
– Flap loss	13 (2.9)
– Partial flap loss	5 (1.1)
– Arterial thrombosis	10 (2.2)
– Venous congestion	15 (3.4)
– Bleeding/Hematoma	15 (3.4)
– Infection	9 (2.0)
– Seroma	1 (0.2)
Minor complication, n (%)	
– Wound dehiscence	12 (2.6)
– Partial flap necrosis	1 (0.2)
– Infection	2 (0.4)
– Seroma	4 (0.9)

### Venous anastomoses

Several variations of venous anastomoses have been performed (Table 3). In addressing whether one or two venous anastomoses (two cranial or cranial and caudal connections to the internal mammary vein) were advantageous or disadvantageous, it was determined that anastomosing two veins did not result in a significant reduction of major complications including complete flap loss (p=0.193), partial flap loss (p=0.485), or venous congestion (p=0.508). In some cases where the internal mammary veins were found to be very small in caliber, alternative recipient veins were used as a precautionary measure to ensure reliable venous outflow (Table 3). This decision was made intraoperatively to reduce the risk of venous congestion and associated complications. In cases where the primary venous anastomosis was not performed using the standard recipient vessels (one or two connections of the deep inferior epigastric vein (DIEV) to the internal mammary vein (IMV)), a significantly higher rate of venous congestion (0.012) and flap loss (p=0.036) was observed.

**Table 3:** Venous anastomoses and complications.

Venous anastomosis	n (%)	Venous congestion, n (%)	Flap loss, n (%)	Partial flap loss, n (%)	Salvage procedure, n (%)
Primary donor and recipient vein					

1x DIEV – 1x IMV cranial	300 (67.1)	7 (2.3)	1 (0.3)	1 (0.3)	3 (1.0)
2x DIEV – 2x IMV cranial	82 (18.3)	2 (2.4)	2 (2.4)	0 (0)	–
2x DIEV – 1x IMV cranial, 1x IMV caudal	36 (8.1)	2 (5.6)	0 (0)	1 (2.7)	2 (5.6)
1x DIEV – 1x IMV, 1x DIEV – 1x cephalic vein	2 (0.4)	0 (0)	0 (0)	0 (0)	–
1x DIEV – 1x subclavian vein	1 (0.2)	0 (0)	0 (0)	0 (0)	–
1x DIEV – 1x cephalic vein	3 (0.7)	1 (33.3)	0 (0)	0 (0)	1 (33.3)
1x DIEV – 1x IMV cranial, 1x SIEV supercharged					
– 1x IMV cranial	3 (0.7)	1 (33.3)	0 (0)	0 (0)	1 (33.3)
– 1x IMV caudal	3 (0.7)	0 (0)	0 (0)	0 (0)	–
– 1x cephalic vein	1 (0.2)	0 (0)	0 (0)	0 (0)	–
SIEV turbocharged (in flap anastomosis) to DIEV					
– 1x DIEV – 1x IMV cranial	5 (1.1)	0 (0)	0 (0)	0 (0)	–
– 2x DIEV – 2x IMV cranial	6 (1.3)	0 (0)	0 (0)	0 (0)	–
2x SIEV – 2x IMV cranial	1 (0.2)	1 (100)	1 (100)	0 (0)	–
1x SIEV – 1x IMV cranial	4 (0.9)	1 (25.0)	1 (25.0)	0 (0)	–

Salvage procedures Secondary donor and recipient vein					

1x DIEV – 1x IMV caudal	1 (14.3)	0 (0)	0 (0)	0 (0)	–
1x DIEV – 1x cephalic vein	2 (28.6)	1 (50.0)	1 (50.0)	0 (0)	–
1x DIEV – vein interposition graft – external jugular vein	2 (28.6)	0 (0)	0 (0)	0 (0)	–
1x DIEV – 1x IMV cranial, 1x SIEV supercharged – 1x intercostal vein	1 (14.3)	0 (0)	0 (0)	0 (0)	–
1x DIEV – 1x contralateral IMV caudal	1 (14.3)	0 (0)	0 (0)	0 (0)	–

DIEV, deep inferior epigastric vein; IMV, internal mammary vein; SIEV, superficial inferior epigastric vein.

**Figure 1:**
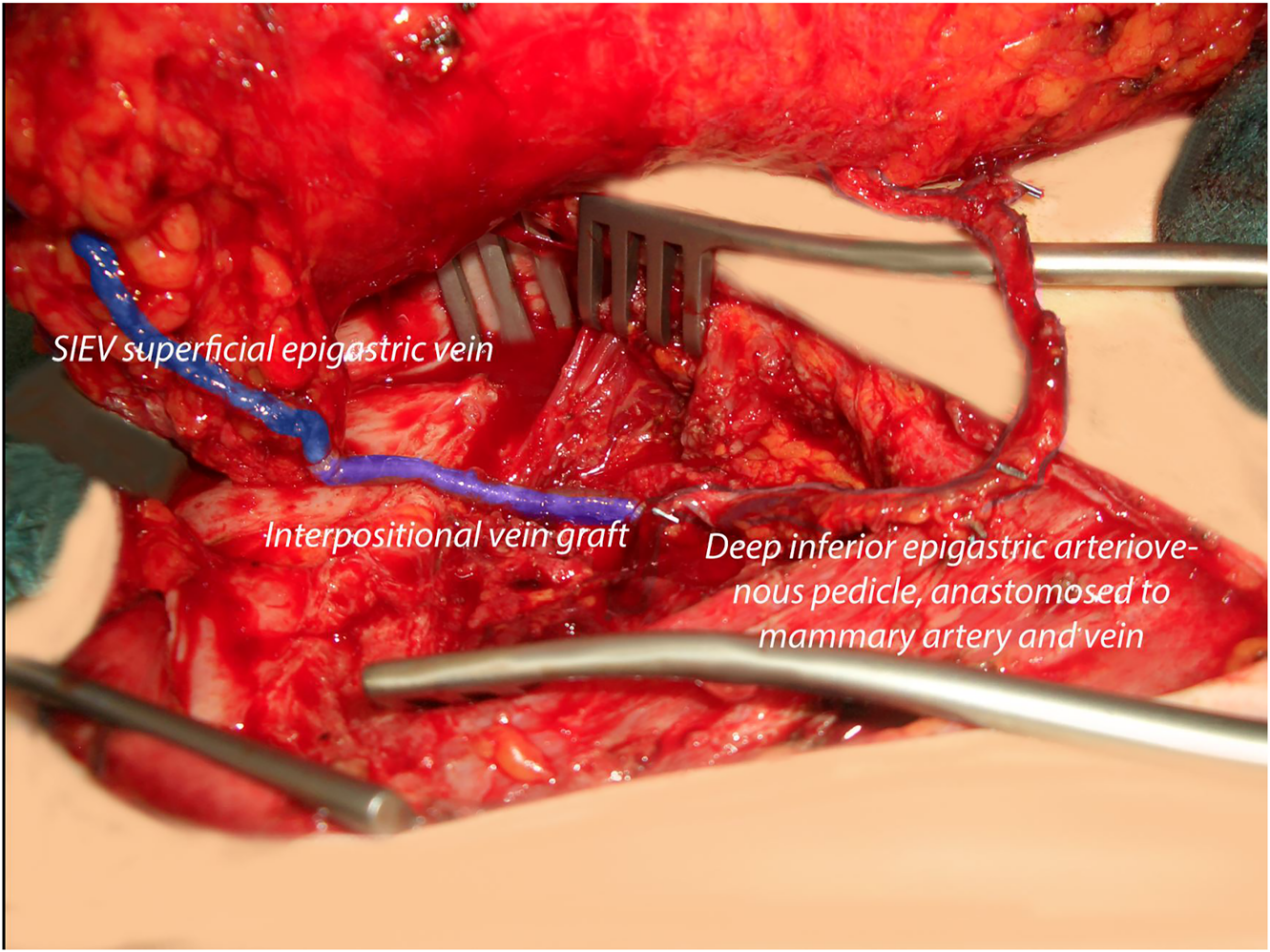
Intraoperative site after interpositional vein graft to enhance outflow from the superficial venous system into the deep inferior epigastric system (modified after [33], copyright REH 2025).

**Figure 2:**
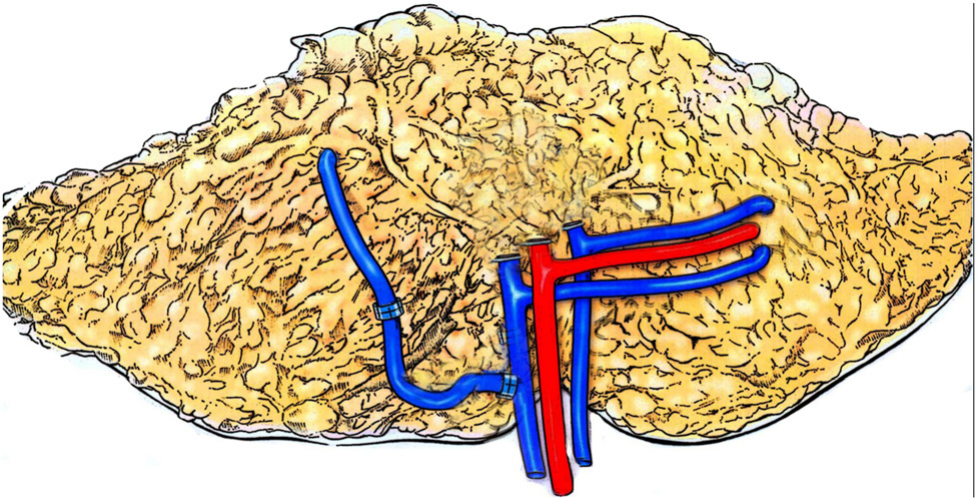
Schematic rendering of interpositional vein graft to enhance venous drainage from the superficial epigastric vein to the deep epigastric vein (copyright REH 2025).

Before the introduction of intraoperative indocyanine green (ICG) angiography, superficial inferior epigastric vein (SIEV)-super- or turbocharging (Figures 1 and 2) was performed intraoperatively in patients with suspected critical venous drainage from the flap. Exclusive anastomosis of the superficial venous system via the SIEV was associated with relatively high complication rates. In contrast, SIEV supercharging or turbocharging have demonstrated very low complication rates with no significant increase in major or minor complications. These findings support the approach that, even in cases where the DIEV appears small or underdeveloped, the deep system should still be used as the primary venous outflow.

In one patient, venous drainage problems intraoperatively necessitated a venous connection to the subclavian vein using an interposition graft. This successfully salvaged the flap, but minor complications occurred (wound healing disorders). In cases of poor vessel quality of the internal mammary vein (IMV), the cephalic vein was used as the primary recipient vein in three patients and as a salvage option in two additional patients. The cephalic vein was skeletonized up to its vascular outlet from the subclavian vein and then tunneled to the flap for anastomosis. Among these five cases, two developed venous congestion and one resulted in total flap loss. These outcomes suggest that the cephalic vein is a less reliable option for venous drainage and should only be considered when no more favorable alternatives are available. Other salvage procedures proved to be more promising, showing better outcomes in terms of venous congestion and flap failure, e.g. anastomosing the DIEV to the IMV caudal, to the external jugular vein via vein interposition graft, or SIEV supercharging.

In one patient that unterwent a bilateral reconstruction, a venous cross-over bypass was performed because of intraoperative inadequate venous drainage. Due to insufficient ipsilateral venous recipient vessels after a thrombosis of the left subclavian port system in her medical history, the venous anastomosis was performed with a pre-sternal, subcunaeously tunneled saphenous vein graft to the contralateral caudal IMV. The patient did not develop any major or minor complications.

### Arterial anastomoses

The analysis of arterial anastomoses revealed individual cases in which the typical vascular connection to the cranial internal mammary artery (IMA) was not feasible due to inflow problems. Alternative connections were only made in exceptional cases, which are listed in Table 4.

**Table 4:** Recipient arteries and complications.

Arterial anastomosis	No. of cases (%)	Arterial thrombosis, n (%)	Flap loss, n (%)	Partial flap loss, n (%)
IMA cranial	432 (96.6)	6 (1.4)	4 (0.9)	0 (0)
IMA caudal	7 (1.6)	0 (0)	0 (0)	0 (0)
IMA cranial + caudal (contralateral DIEA)	3 (0.7)	0 (0)	0 (0)	0 (0)
Subclavian artery via saphenous vein interposition graft	3 (0.7)	1 (33.3)	1 (33.3)	0 (0)
Thoracoacromial artery via vein interposition graft	2 (0.4)	2 (100)	2 (100)	0 (0)

IMA, internal mammary artery; DIEA, deep inferior epigastric artery.

A caudal anastomosis to the IMA was performed in only n=7 patients. No major complications were observed and no statistically significant difference to the cranial anastomosis was found (p>0.999). This indicates that, in exceptional cases, an anastomosis to the caudal IMA might be a safe option that ensures adequate flap perfusion. In two patients, the contralateral deep inferior epigastric artery (DIEA) was also harvested, allowing for two aterial anastomoses to be performed – one cranially and one caudally – on the IMA. These flaps also proceeded without complications. In three cases, an arterial connection to the subclavian artery using a saphenous vein interposition graft was necessary. Two of the three patients developed major complications, including arterial revisions, postoperative bleeding, and in one case complete flap loss. Additionally, in one case a conservatively treated wound healing disorder was observed.

In one case, the arterial connection was made to the thoracoacromial artery using a saphenous vein interposition graft. While the immediate postoperative course showed normal flap perfusion with patency of the anastomoses, a secondary flap loss occurred one week later. A subsequent arterial revision was unsuccessful. In another case, the anastomosis was performed with a cephalic vein interposition graft to the thoracoacromial artery. After initially showing regular perfusion, a hematoma and arterial thrombosis occured three days postoperatively, so that the flap had to be removed. So far, this salvage option does not appear to be promising. However, due to the low number of cases, no definitive conclusions can be drawn.

### Previous breast surgeries

After prior mastectomy (ME) and expander or prosthesis implantation, no higher rate of major (p=0.632) or minor complications (p=0.086) was observed. In particular, the occurrence of seromas or wound healing disorders was not observed more frequently after expander and/or prosthesis implantation (seromas: n=1 (0.6 %); wound healing disorders: n=2 (1.3 %)) than after mastectomy alone or mastectomy and previous flap plasty (seromas: n=3 (1.0 %); wound healing disorders: n=10 (3.5 %)).

### Radiotherapy

In our cohort, radiotherapy was administered prior to n=262 (59 %) of the flap reconstruction procedures. A targeted analysis of the correlation between flap revisions and previous radiotherapy for breast cancer showed a significantly increased postoperative complication rate in patients with prior radiation therapy. The number of required arterial flap revisions was significantly higher in this patient group compared to those without prior radiation therapy (p=0.0415). A significantly increased flap loss rate or significantly increased venous stasis was not observed after irradiation. No correlation was found between SIEV turbocharging and prior radiotherapy. Notably, considering minor complications, all documented breast seromas (n=4) and infections (n=2) occurred in patients who had undergone radiotherapy, whereas no seromas or infections were observed in patients without prior radiation exposure. However, due to the small number of cases, this difference cannot be considered statistically significant.

## Discussion

Autologous microsurgical breast reconstruction has been established as one of the most effective methods for restoring the breast following mastectomy for breast cancer [1], [12], [13], [14]. Compared to implant-based procedures, it offers numerous advantages, particularly a more natural appearance, better tissue compatibility, and long-term stability of the result [14]. However, this complex surgical procedure remains associated with various pre-, intra-, and postoperative challenges, which significantly impact the success of reconstruction.

Overall, vascular anastomotic issues remain a key challenge and are the most common complications in microsurgical tissue transplantation [8], 9]. Anastomotic failures, thrombosis, or insufficient venous outflow can lead to partial or complete flap loss. Alongside advancements in microsurgery and the expansion of indications, a wide variety of techniques have been introduced to improve venous drainage and address the issue of venous congestion [15], [16], [17], [18]. In our cohort, flap loss was significantly more common when standard venous anastomosis to the IMV was not used, underscoring their role as the preferred recipient vessels. However, in certain cases, alternative venous anastomoses may still be necessary as a salvage option to preserve the flap when standard recipient vessels are not suitable or available. Despite relatively large patient cohorts from different centers, the clinical benefit of various techniques in salvaging compromised flaps has not yet been widely demonstrated due to the relatively low number of salvage procedures reported in the literature. Enajat et al. described a significant reduction of venous congestion by using a secondary vein in the drainage of a DIEP flap [19]. In our study, a double anastomosis of the cranial IMV (medial and lateral) or cranial and caudal IMV was not associated with a reduction in overall major complications, venous congestion, flap loss, or postoperative bleeding and hematomas. Sulli et al. reported venous compromise in 16 out of 101 free flaps; among these, 11 flaps (13.2 %) were in the single-vein group, and five flaps (27.7 %) were in the two-vein group [20]. While their study showed that re-exploration rates were lower in the single-vein group compared to the two-vein group, this difference was not statistically significant. However, they described an improved rate of flap salvage with two-vein anastomosis. Their findings suggest that a single venous anastomosis may be sufficient for a successful free flap transfer. Similarly, Kahn et al. found no difference in flap survival rates between single- and two-vein anastomoses, but observed higher re-exploration rates in the two-vein group. However, they described better salvage rates in the two-vein group than in the single-vein group [21]. On the contrary, other studies, however, suggest that perforator flaps with a single venous anastomosis may require more frequent revision surgeries compared to those with two venous anastomoses [22]. Pozzo et al. reported that venous congestion due to superficial venous dominance (SVD) occurs in approximately 2 % of cases in DIEP flap procedures, leading to postoperative complications [23]. These authors analyzed 24 patients in the SVD group and found that computed tomography angiography (CTA) imaging revealed a significantly higher SIEV/DIEV diameter ratio in the SVD group compared to the control group. They concluded that the SIEV/DIEV ratio on preoperative CTA could predict whether a DIEP flap exhibits SVD. Based on this information, the need for an additional venous anastomosis could be anticipated to reduce postoperative complications. In our patient cohort, reliable flap perfusion was achieved through the application of both supercharging and turbocharging techniques, demonstrating the safety of these technqiues. Pignatti et al. analyzed venous supercharging in case of venous congestion and showed a statistically significant advantage of super-drainage to reduce the venous congestion of the flap, partial flap necrosis, total flap necrosis, and flap revision [24]. Another rescue option in the event of insufficient venous outflow via the ipsilateral IMV is the cross-over venous emergency bypass to the contralateral IMV, which we described as a useful tool in unexpected venous thrombosis during bilateral free flap breast reconstruction [16].

In our patient cohort, connecting the vena epigastrica to the vena cephalica was associated with an increased need for venous flap revisions and might only be performed after other rescue options have been exhausted.

In terms of arterial anastomoses, the results of this study suggest that, in exceptional cases, a caudal anastomosis to the IMA may be a viable and safe alternative, as demonstrated by the absence of major complications in the seven patients who underwent this procedure. Jaiswal et al. performed a retrospective study of microvascular breast reconstruction using retrograde internal mammary donor vessels [25]. They described a total of 35 cases, of which the caudal internal mammary vessels were used for the solitary set of microvascular anastomoses and observed no flap loss. According to our findings, Jaiswal et al. suggested that the distal end of IMA and IMV on retrograde flow might be safe as an additional or sole pedicle. This allows for the preservation of other options, such as the thoracodorsal pedicle and latissimus dorsi flap, for use in the event of complications or recurrence.

The use of saphenous or cephalic vein interposition grafts for anastomosis to the subclavian and thoracoacromial arteries has been explored as a salvage option in cases where the IMA is unsuitable or unavailable. In our cohort, this was associated with an increase in major complications, including flap loss and postoperative bleeding, as could be expected. However, it must also be considered that without these revisions, none of these flaps could have been saved. The use of venous interposition grafts is generally described as problematic [26], and the length of a venous interposition graft is considered more critical than that of an arterial interposition graft [27]. Further refinements and a better understanding of flow dynamics in microvascular anastomoses are expected through intraoperative blood flow measurements before and after anastomosis [28]. Based on our experience, arterial inflow from the contralateral internal mammary artery can also successfully maintain flap perfusion in doubtful cases [18].

Our findings demonstrate that prior radiation therapy is associated with an increased rate of postoperative complications, particularly in the need for arterial flap revisions. Radiation therapy can lead to fibrosis and vascular damage [29], which might challenge anastomosis and increase the risk of delayed wound healing and flap failure. However, there were no higher rates of flap loss or partial flap loss in women with a history of radiation therapy in our cohort. This is in accordance with the findings of Prantl et al. [30]. However, they described a higher risk for wound healing disorders after irradiation. Whereas we found no higher rates of wound healing disturbances, all recorded cases of breast seromas and infections occurred in patients with prior radiation therapy, while no such complications were observed in non-irradiated patients. Although the low number of cases prevents statistical significance, these findings are consistent with studies indicating that radiation induces microvascular damage [29], which might enhance the risk of infection due to reduced tissue perfusion.

Our results indicate that prior ME and expander or prosthesis implantation did not lead to a significantly higher rate of major or minor complications in subsequent autologous breast reconstruction. These findings align with previous studies that reported similar flap success rates and complication profiles in patients transitioning from implant-based to autologous reconstruction compared to those undergoing primary autologous reconstruction [31], 32].

This study has certain limitations, primarily due to its retrospective design. The rarity of vascular complications, and consequently the limited cases of rescue maneuvers, often prevents the drawing of statistically reliable conclusions. In addition, the infrequent occurrence of some complications in both comparable groups increases the risk of confounding variables and bias in these cases. Another limitation is the potential for bias due to the long study period, during which gradual advancements and individual surgeons’ learning curves may have influenced outcomes. Nevertheless, the data were collected from an academic teaching institution with standardized protocols during procedures and in postoperative care, and all participating surgeons were highly experienced microsurgeons. Our findings highlight the importance of a differentiated approach in microsurgical breast reconstruction planning. Optimizing vascular anastomosis techniques, as well as thorough evaluation of prior treatments including radiotherapy and soft tissue quality, are essential to minimizing major and minor complications. Given these potential complications, careful preoperative planning, consideration of individual risk factors, selection of the appropriate vascular anastomosis, and structured postoperative monitoring are essential to minimizing complications and achieving optimal reconstructive outcomes [4]. Further studies are needed to better characterize patient-specific risk factors and develop personalized treatment strategies. To further improve outcomes in autologous breast reconstruction – particularly in complex cases with non-standard recipient vessels – future research should focus on developing evidence-based guidelines through prospective, multicenter trials. Imaging-based risk stratification using preoperative modalities such as CTA or MRA may help identify patients at higher risk for vascular complications and guide recipient vessel selection. In addition, the routine use of intraoperative perfusion assessment tools, such as ICG angiography, could support real-time decision-making and reduce flap-related complications. These strategies would allow for more personalized surgical planning and optimize reconstructive outcomes.

## Conclusions

Vascular anastomosis selection correlates with major and minor complication rates. Both arterial and venous anastomosis techniques, including preventive measures such as supercharging, can lead to improved outcomes. Our study confirms that radiotherapy is a significant risk factor for postoperative complications, requiring an adjusted surgical strategy. Further research in high-volume centers is needed to refine techniques for specific flap reconstructions and better address patient-specific perioperative risks.
